# Allo Beta Cell transplantation: specific features, unanswered questions, and immunological challenge

**DOI:** 10.3389/fimmu.2023.1323439

**Published:** 2023-11-23

**Authors:** Rossana Caldara, Valentina Tomajer, Paolo Monti, Valeria Sordi, Antonio Citro, Raniero Chimienti, Chiara Gremizzi, Davide Catarinella, Stefano Tentori, Vera Paloschi, Raffella Melzi, Alessia Mercalli, Rita Nano, Paola Magistretti, Stefano Partelli, Lorenzo Piemonti

**Affiliations:** ^1^ Clinic Unit of Regenerative Medicine and Organ Transplants, IRCCS Ospedale San Raffaele, Milan, Italy; ^2^ Pancreatic Surgery, Pancreas Translational & Clinical Research Center, IRCCS Ospedale San Raffaele, Milan, Italy; ^3^ Diabetes Research Institute, IRCCS Ospedale San Raffaele, Milan, Italy; ^4^ Università Vita-Salute San Raffaele, Milan, Italy

**Keywords:** islet transplant, immunosuppression, type 1 diabetes, autoimmunity, beta cell replacement, immunomodulation

## Abstract

Type 1 diabetes (T1D) presents a persistent medical challenge, demanding innovative strategies for sustained glycemic control and enhanced patient well-being. Beta cells are specialized cells in the pancreas that produce insulin, a hormone that regulates blood sugar levels. When beta cells are damaged or destroyed, insulin production decreases, which leads to T1D. Allo Beta Cell Transplantation has emerged as a promising therapeutic avenue, with the goal of reinstating glucose regulation and insulin production in T1D patients. However, the path to success in this approach is fraught with complex immunological hurdles that demand rigorous exploration and resolution for enduring therapeutic efficacy. This exploration focuses on the distinct immunological characteristics inherent to Allo Beta Cell Transplantation. An understanding of these unique challenges is pivotal for the development of effective therapeutic interventions. The critical role of glucose regulation and insulin in immune activation is emphasized, with an emphasis on the intricate interplay between beta cells and immune cells. The transplantation site, particularly the liver, is examined in depth, highlighting its relevance in the context of complex immunological issues. Scrutiny extends to recipient and donor matching, including the utilization of multiple islet donors, while also considering the potential risk of autoimmune recurrence. Moreover, unanswered questions and persistent gaps in knowledge within the field are identified. These include the absence of robust evidence supporting immunosuppression treatments, the need for reliable methods to assess rejection and treatment protocols, the lack of validated biomarkers for monitoring beta cell loss, and the imperative need for improved beta cell imaging techniques. In addition, attention is drawn to emerging directions and transformative strategies in the field. This encompasses alternative immunosuppressive regimens and calcineurin-free immunoprotocols, as well as a reevaluation of induction therapy and recipient preconditioning methods. Innovative approaches targeting autoimmune recurrence, such as CAR Tregs and TCR Tregs, are explored, along with the potential of stem stealth cells, tissue engineering, and encapsulation to overcome the risk of graft rejection. In summary, this review provides a comprehensive overview of the inherent immunological obstacles associated with Allo Beta Cell Transplantation. It offers valuable insights into emerging strategies and directions that hold great promise for advancing the field and ultimately improving outcomes for individuals living with diabetes.

## 1 Introduction: “Beta is better.”

### 1.1 Despite the availability of insulin therapy, T1D patients face challenges in achieving optimal blood sugar control, chronic complications, and mental health burden

In 2022, around 8.75 million people with T1D were living with the condition, with 1.52 million under 20 (IDF Diabetes Atlas 10th edition, https://diabetesatlas.org/). In the early 20th century, T1D was often fatal, with children dying within a short time after diagnosis (1). The discovery of insulin by Banting, Best, Collip, and Macleod in 1921 revolutionized diabetes care, offering hope to countless individuals (2). Leonard Thompson became the first T1D patient to receive insulin, marking the beginning of a century of innovations in diabetes treatment. Over time, the treatment goal for T1D has shifted from merely keeping patients alive to achieving nearly normal blood sugar levels. While insulin was once seen as a highly effective treatment, it is now recognized as insufficient, as it transforms a fatal condition into a chronic and degenerative disease (3). Healthy individuals maintain blood glucose levels close to 99 mg/dL on average, with minimal variability (4). Even with advanced technologies like closed-loop systems (5) and adjunctive therapies (i.e., SGLT-2 inhibitor, low-carb diet), T1D patients struggle to achieve these levels (6–8). Current consensus guidelines define a target range of 70-180 mg/dL (9, 10), which still falls far from healthy norms (11). Maintaining blood sugar levels as close to normal as possible is essential (12–19). Lowering blood sugar levels is associated with reduced risks of complications and mortality in T1D (20–22). A 1% reduction in HbA1c has been linked to decreased risks of myocardial infarction, stroke, microvascular complications, and more (23). Despite the availability of advanced treatments, a substantial proportion of T1D patients fail to meet glycemic targets (24). Registries and clinics report that many children, adolescents, and adults do not achieve HbA1c goals (25–28). Even with the use of technology (5), blood sugar control remains elusive (6–8). Patients with T1D face acute complications related to insulin therapy, including hypoglycemia (29–33). Hypoglycemia rates remain significant, impacting patients’ cognitive function (34–38), cardiovascular health (39–42), and quality (43, 44) and quantity of life. Chronic complications continue to develop, despite advances in insulin and devices, affecting kidney function, retinopathy, and more. Insulin therapy can also have a significant negative impact on mental health, contributing to diabetes distress (45). Approximately 20-30% of T1D individuals experience this burden, which persists even with new technologies (46, 47). While there has been a decline in T1D-related mortality (48, 49), it still presents a significant risk. Patients with T1D face a relative risk of mortality 3.1 to 5.8 times higher than those without diabetes (North America (50, 51), Europe (52–54) and Australia (55)). This results in an estimated loss of 10 to 13 years of life (54–57). In conclusion, insulin therapy has undeniably been a life-saving treatment for individuals with T1D. However, it falls short of providing a normal and healthy life. Achieving optimal blood sugar control remains a significant challenge, and chronic complications continue to be a concern. Moreover, the psychological toll of managing T1D cannot be overlooked.

### 1.2 Pancreas and islet transplantation are effective treatments for T1D, improving glycemic control and life expectancy

As we commemorate a century of insulin discovery two years ago, it is imperative to renew our commitment to finding more effective treatments and ultimately striving for a world where individuals with T1D can live without the constraints of insulin therapy (3). Over the past three decades, clinical trials have demonstrated that restoring beta-cell function through islet or pancreas transplantation can lead to more physiologic regulation of blood sugar levels compared to exogenous insulin in diabetes patients (58). Clinical trials are essential for evaluating the safety and efficacy of new treatments, and they have played a vital role in the development of islet transplantation for T1D. Four successful large-scale Phase 3 clinical trials in islet transplantation have been published recently: CIT-07 (multicenter, single-arm) (59), CIT06 (pivotal trial) (60), TRIMECO (multicenter, open-label, randomized) (61) and REP0211 (multicenter, Double blind, randomized) (62). All these studies have provided compelling evidence that the transplantation of human islets into patients with T1D who experience impaired awareness of hypoglycemia and severe hypoglycemic events is not only safe but also highly effective in maintaining optimal glycemic control (3). Attaining freedom from the need for insulin can be realized by transplanting a sufficient quantity of islets (63). Following islet transplantation, the likelihood of sustaining insulin independence for up to five years may reach as high as 50%. Furthermore, a substantial proportion of patients, approximately one in four, may continue to be insulin independent, maintaining HbA1c levels at or below 6.5%, for a period spanning at least a decade. This favorable outcome can be achieved through either islet transplantation as a standalone procedure or in conjunction with a kidney transplant (64, 65). The glucose control achieved with excellent islet graft function closely resembles glucose values observed in healthy adults, with median glucose levels at 103 mg/dl, a standard deviation around the mean value of 14, and no time spent above 180 mg/dl or below 54 mg/dl. HbA1c levels typically fall within the range of 5.6 to 5.8 (66). Moreover, standardized psychometric instruments and psychologist-conducted interviews have confirmed a significant improvement in the quality of life following islet transplantation (67–75). Additionally, there is evidence of positive effects on the microvascular complications of T1D, including the stabilization or slower progression of retinopathy (76–79) and neuropathy (77, 80–82), as well as improvements in micro- and macroangiopathy (74, 76, 83–91). Pancreatic transplantation, in conjunction with islet transplantation, stands as the other effective treatment option for reinstating normal glycemic control in individuals with T1D (92). Simultaneous pancreas-kidney (SPK) transplantation is the most commonly performed type of pancreas transplantation (93), primarily T1D patients with end-stage renal failure. After more than five decades of worldwide experience and over 80,000 reported cases to the International Pancreas Transplant Registry, there is substantial evidence demonstrating that SPK transplantation enhances life expectancy (94–96) and mitigates the progression of diabetic complications (97–99). Similarly, sequential pancreas after kidney (PAK) transplantation, whether following a living or deceased donor kidney transplant, has shown improvements in long-term patient and kidney graft survival rates (100). Pancreas transplantation alone (PTA) is also considered a rational therapy for appropriately selected T1D patients experiencing life-threatening metabolic complications (101–104).

### 1.3 Clinical trials using stem cell-derived islet cells for the treatment of T1D are ongoing, with promising preliminary results

The field of cellular therapies for the treatment of T1D is rapidly evolving and a new exciting era has already begun. Human pluripotent stem cells, including both embryonic stem (ES) and induced pluripotent stem (iPS) cells, are considered the most promising candidates for generating β cells due to their capacity for unlimited growth and differentiation. Multiple laboratories have developed effective protocols for differentiating these pluripotent cells into β cells, focusing on producing cellular products that are consistently potent and safe for clinical use (105–113). Currently, there are nine clinical trials registered in ClinicalTrial.gov utilizing human pluripotent stem cells for the treatment of T1D (NCT04678557, NCT02939118, NCT03162926 NCT02239354, NCT03163511 NCT05210530, NCT05565248, NCT04786262, NCT05791201). Three of these trials are active and recruiting patients, two have been completed, one was terminated, and three are active but not recruiting. Seven trial are using pancreatic precursor cells (PEC-01) derived from pluripotent stem cells (genetically modified in two trials, PEC211) in combination with durable, removable, close or perforated devices (114). These cells are a mixed population of pancreatic precursor cells (73%–80%NKX6.1^+^/PDX1^+^ pancreatic precursor) fully committed to further differentiating into mature endocrine pancreatic cells (115) once implanted within an encapsulation device in a subcutaneous space. Interim results from some of these clinical trials, reported in December 2021, were promising but not yet clinically meaningful. Over a follow-up period of up to 1 year, patients experienced a 20% reduction in insulin requirements, spent 13% more time within the target blood glucose range, maintained stable average HbA1c levels below 7.0%, and improved hypoglycemic awareness. Additionally, C-peptide levels, a marker of insulin production, were detected at approximately 1/100th of normal levels within explanted grafts, which included various types of pancreatic cells, including cells with a mature β cell phenotype. The immunosuppressive treatment appeared effective in preventing graft rejection, and the cell product demonstrated safety and tolerability, with no teratoma formation observed (116, 117). In 2021, VX-880, an investigational cell therapy for T1D, was approved as a second cell product. VX-880 comprises fully differentiated insulin-producing pancreatic islet cells derived from pluripotent stem cells. A Phase 1/2 clinical trial was approved for patients with T1D who have impaired hypoglycemic awareness and severe hypoglycemia. VX-880 is administered through infusion into the portal vein, and concomitant immunosuppressive therapy is necessary to protect the islet cells from immune rejection. Preliminary results suggest that β cells derived from stem cells and transplanted into the liver can engraft and begin secreting insulin shortly after infusion and provide insulin independence in patients with T1D (118). In addition to the ongoing clinical efforts, several commercial and academic organizations have announced their plans to conduct clinical trials using functional stem cell-derived islets.

### 1.4 Allo Beta Cell transplantation offers hope for a cure for T1D, but further research is needed to address the challenges of long-term immunosuppression and graft rejection

Allo Beta Cell transplantation is a promising cure for T1D, but it is not yet a widely available option because it requires patients to take lifelong immunosuppressive drugs. These drugs have serious side effects, including an increased risk of infection and cancer. Therefore, it is important to carefully weigh the risks and benefits of Allo Beta Cell transplantation for each individual patient. Factors to consider include the severity of T1D, the risk of complications from chronic immunosuppression, the patient’s willingness to comply with treatment, and their life expectancy. Allo Beta Cell transplantation may be a good option for people with severe T1D or a high risk of complications, or for people who have tried other treatments without success. Researchers are working on ways to protect transplanted beta cells from immune rejection without the need for chronic immunosuppression. This would make Allo Beta Cell transplantation a more viable option for a wider range of people with T1D. One promising approach is to use encapsulation devices. Encapsulation devices protect transplanted beta cells from the immune system by enclosing them in a semipermeable membrane. This allows the beta cells to secrete insulin into the bloodstream, but it prevents the immune system from attacking them. Another promising approach is to use gene editing to modify the beta cells before transplantation. This could make them less susceptible to attack by the immune system. Researchers are also working to develop new immunosuppressive drugs that are more effective and have fewer side effects. These advances could make Allo Beta Cell transplantation a safe and effective cure for T1D in the near future.

## 2 Immunological specific hallmark in Allo Beta Cell transplantation

Allo Beta Cell Transplantation presents distinct immunological hurdles when compared to the transplantation of other organs or tissues. These challenges are primarily associated with the unique functions and biology of beta cells, the site of infusion, and the individual characteristics of the recipient.

### 2.1 The importance of glucose regulation and insulin in immune activation

The regulation of glucose levels and the presence of insulin are pivotal factors in immune activation (119). A significant association between post-transplant glycemic control and the development of subsequent rejection was reported for solid organ transplantation (120–122). In contrast to other transplanted organs, beta cells are responsible for producing insulin and maintaining glucose equilibrium. Consequently, in Allo Beta Cell Transplantation, the effectiveness of the graft is also crucial for the immunological response.

#### 2.1.1 Insulin and immunity

Insulin, a key hormone in glucose metabolism, also has immunomodulatory effects, promoting both pro- and anti-inflammatory responses in a variety of immune cells (122, 123). In macrophages and neutrophils, insulin activates insulin receptors (InsR) and insulin-like growth factor 1 receptors (IGF1R), which triggers signaling pathways that lead to the production of pro-inflammatory cytokines, chemokines, and adhesion molecules (123, 124). Insulin increases the production of reactive oxygen species (ROS), which can activate pro-inflammatory signaling pathways and produce pro-inflammatory mediators (125, 126). Insulin also promotes the activation and survival of eosinophils (127), the maturation and scavenger receptor expression of dendritic cells (128), and the activation, cytokine production, and differentiation of natural killer (NK) cells and innate lymphoid cells (ILCs) (129). In adaptive immunity, insulin predominantly assumes a pro-inflammatory role by optimizing T cell activation, enhancing their responsiveness to key cytokines, and facilitating migration to sites of infection or inflammation (130–134). T cells without InsR have metabolic and functional problems, resulting in less production of important immune molecules, such as IFNγ, and impaired expansion in response to specific antigens (135). In addition, InsR signaling seems to affect the balance of regulatory T cells (Tregs) in the immune system, which could have implications for conditions where insulin signaling weakens the suppressive function of Tregs (136). T cells also express the IGF1R, which plays a role in regulating the differentiation of T helper 17 (Th17) cells and Tregs (137). The precise influence of IGF1R signaling on these processes depends on contextual factors, such as the differentiation stage of the T cells and the presence of specific ligands. In B cells, while the exact role of InsR signaling remains less clear, it is known that B cells express InsR (138). Elevated local and systemic insulin levels are common in patients who have received islet transplants, due to the production of insulin by the transplanted islets and the need for supplemental insulin therapy. Elevated insulin levels may contribute to the risk of inflammation and rejection, as shown in one study that found a higher risk of islet graft dysfunction in patients with higher insulin levels (139) and further sustained by our recent study reporting that progression to Stages 2 and 3 of T1D increases with HOMA-IR and decreases with the Matsuda Index (140).

#### 2.1.2 Glucose and immunity

Glucose metabolism plays a central role in supporting the functions of innate immune cells (141). High glucose levels can induce the production of ROS (142), which can serve as potent weapons against invading pathogens but can also lead to oxidative stress and inflammation (143). Additionally, high glucose levels can upregulate inflammatory cytokines and chemokines, activate NF-κB, PKC, and p38 MAPK pathways, and alter T-cell activation, differentiation, and functions (144, 145). While existing evidence suggests that persistent high blood glucose levels can induce notable molecular and functional alterations in T cells, resulting from shifts in their proteomic and metabolic profiles (146), it’s worth noting that short term elevated blood glucose levels may actually enhance immune responses (147). Additionally, hyperglycemia prompts CD4 T cells to adopt an activated immunophenotype (148). In line with these findings, high blood glucose levels during and after kidney and liver transplantation are associated with higher rates of organ rejection (119). In a study of mice, the timing of islet allograft loss was dependent on the degree of hyperglycemia in the recipient (149). Hyperglycemia is common in islet transplant patients for s reasons, including the underlying diabetic condition, difficulty controlling blood sugar levels before transplantation, and medications and infections that can occur after transplantation. Hyperglycemia can increase the risk of rejection, so it is important to carefully manage blood glucose levels in these patients.

### 2.2 The importance of beta cell in the interaction with immune cells

In recent years, significant advancements have emerged in our ability to comprehensively study the interaction between beta cells and immune cells (150). Notably, recent research has reshaped our understanding, highlighting that pancreatic beta cells play an active role rather than remaining passive during the progression of immune recognition (151). It was previously believed that T1D resulted in the complete depletion of beta cells. However, recent studies have uncovered a distinct subset of beta cells that manage to survive, although their functionality is limited (152–154). This revelation suggests that not all beta cells are equally susceptible to immune responses, potentially due to inherent protective mechanisms. As scientific inquiries have revealed a wide spectrum of variations among beta cells (155–158), encompassing genetic expression, physical characteristics, functionality, and communication with neighboring cells, this diversity implies that the unique traits of beta cells themselves could influence their capacity to withstand immune attacks (159). Another intriguing development gaining recent attention proposes that specific stress events affecting beta cells can trigger immune cell activation (160). These pathways include inflammatory stress originating from both innate and adaptive immune responses, as well as endoplasmic reticulum (ER) stress that persists due to the demands of insulin production and intensifies as beta cell mass declines (161, 162). Both pathways are significantly represented in all allogenic beta cell transplantation strategies. The downstream consequences of intrinsic (e.g., ER stress) and extrinsic stressors (e.g., cytokine exposure) on beta cells encompass broad changes in their transcriptomes and proteomes, which can affect the interaction between beta cells and immune cells in a number of ways, including altered expression of surface proteins, secretion of cytokines and chemokines, and changes in metabolic pathways. These changes can alter how they engage with and are perceived by immune cells. For example, a stressed microenvironment plays a crucial role in triggering the overexpression of HLA class I molecules on insulin-producing beta cells (163) and in producing new epitopes (164) formed through various processes, including transpeptidation, disulfide bond formation, deamidation, and citrullination formation of epitopes such as hybrid insulin peptides, alternative splicing, splice variant peptides, and defective ribosomal insulin products (165, 166). Immune recognition of these neoepitopes may be enhanced compared to their native counterparts due to altered HLA binding or increased TCR recognition (167). Adding further complexity to the story, it is now evident that certain gene variants modulate beta cell stress responses, increasing the interindividual variability in how they respond (168–172). Collectively, this evidence suggest that beta cell can be presented to the immune system in a highly individualized and heterogeneous manner, making it difficult to predict and manage the recipient’s immune response to the transplanted beta cells.

### 2.3 The influence of liver site and its significance in the context of immunological challenges

Currently, the liver is the preferred location for clinical Allo Beta Cell Transplantation, despite recent suggestions of alternative implantation sites that might be more advantageous for graft survival (173–181). The intrahepatic site offers benefits: it is a well-established procedure accepted by regulatory agencies and associated with minimal morbidity and a negligible risk of adverse events, such as bleeding and portal thrombosis. Moreover, it allows for the infusion of a substantial tissue volume, up to 20 ml. This site scatters the cells throughout hepatic sinusoids, preventing the formation of clusters that can impede the initial diffusion of oxygen and nutrients. Additionally, it appears to have some immunoprivileged characteristics compared to other sites like the bone marrow and kidney capsule (177, 182, 183). Since the liver is the primary target organ for insulin, intrahepatic islets can mimic physiological pancreatic insulin secretion rather than causing systemic insulin release (184, 185) although there have been suggestions of potential dysfunctional alpha cell function (186). However, the liver presents specific immunological challenges. Monitoring through imaging techniques is not feasible, and routine biopsies are impossible to obtain (187), making it impossible to diagnose rejection promptly. Being an intravascular transplantation, it is prone to the instant blood-mediated inflammatory reaction (IBMIR), an innate immune response that occurs when pancreatic cells encounter ABO-compatible blood. This reaction leads to the release of tissue factor, which activates the coagulation and complement cascades, resulting in leukocyte and macrophage-mediated islet cell death (188–191). Moreover, compared to the native tissue oxygen tension of islets (40 mmHg) and the parenchymal oxygen tension (30 mmHg), the liver provides significantly lower tensions, less than 10 mmHg for both (192) inducing beta cell stress. Amyloid formation (193), associated with type 2 diabetes, has been observed in intraportal islet grafts, and glucolipotoxicity from surrounding hepatocytes has been shown to harm transplanted beta cell (194). Lastly, the liver’s endogenous immune system, including Kupffer cells, Liver sinusoidal endothelial cells, Hepatic stellate cells, Resident liver lymphocytes NK, NKT, and CD8+ T cells, and to a lesser extent, CD4+ T cells), and liver dendritic cells, has also been shown to potentially harm allograft survival at this site (195). As an alternative to liver transplantation, subcutaneous transplantation has emerged as an attractive option for Allo Beta Cell Transplantation. This approach offers advantages, including a straightforward surgical procedure, minimal surgical risks, ease of monitoring, and the potential for graft retrieval. However, its efficacy is hampered by the limited blood supply in the subcutaneous space, which leads to insufficient oxygen and nutrient availability. To overcome these challenges and achieve successful subcutaneous transplantation, a comprehensive approach is essential. This approach involves the integration of bioengineering devices, specialized biomaterials, drug delivery systems, and strategies aimed at promoting early angiogenesis. These components play a crucial role not only in facilitating the incorporation of transplanted insulin-producing cells but also in attaining normoglycemia in recipients. A pivotal aspect of the subcutaneous transplantation’s success lies in the development of biomaterials, including hydrogels derived from both natural polymers (such as collagen, fibrin, and alginate) and synthetic polymers (such as polyethylene glycol and polyvinyl alcohol). These biomaterials can be precisely tailored to possess specific mechanical, biological, and biochemical properties. Importantly, they should exhibit pro-angiogenic properties, fostering the formation of blood vessels within the subcutaneous tissue. These biomaterials can be employed in many ways, serving as coatings for islets or forming the basis for implantable bulk scaffolds. Despite promising advancements in subcutaneous transplantation, challenges persist, particularly when using macro and micro devices for Allo Beta Cell encapsulation. Immune and fibrotic responses can encapsulate these devices, limiting the supply of oxygen and nutrients to the transplanted tissue. Clinical studies employing such strategies have not definitively demonstrated superior long-term outcomes compared to intraportal transplantation. The subcutaneous immune response can often lead to fibrotic overgrowth, adversely affecting islet function. Furthermore, immune-protective devices that physically separate islets from immune cells may underestimate the impact of diffusible immune factors on islet functionality (196). When considering other potential transplantation sites, it is worth noting that the testis, thymus, and the anterior chamber of the eye are regarded as immunoprivileged sites and have been explored as locations for allografts or xenografts. However, they typically cannot accommodate a sufficient number of islets to achieve euglycemia (197).

### 2.4 Recipient and donor matching and their significance in the context of immunological challenges: multiple islet donor preparations and recurrence of autoimmunity

The transplantation of allogeneic beta cells presents specific challenges in adaptive immunology that differ from those encountered in other types of organ or tissue transplants (198). In addition to the innate immune response and issues related to engraftment, transplanted allogeneic beta cells face recognition and rejection by the recipient’s immune system, which is further complicated by the recurrent autoimmune responses in individuals with T1D due to preexisting adaptive immune memory. It is challenging to separate and assess the individual impact of these two phenomena (199). One way to gauge the significance of allorecognition is by evaluating the effect of HLA matching on graft outcomes, as the degree of HLA mismatches correlates with the strength of the immune system’s response. However, the impact of HLA matching on pancreas transplant outcomes remains a topic of debate (200–205). Allogenic immune recognition may be more relevant in the context of islet transplantation, which presents a unique paradigm in organ transplantation due to its requirement for multiple donors to achieve complete insulin independence. Consequently, HLA matching for islets is often minimal, except for the avoidance of preformed anti-HLA antibodies. Some evidence suggests that HLA-A, -B, and -DR matching (excluding HLA-DR3 and -DR4 matching) is associated with improved islet allograft survival (206–208). Regrettably, due to the more stringent donor selection criteria in islet transplantation relative to other transplant procedures and the substantial risk of manufacturing failures, achieving HLA matching is scarcely feasible in clinical practice. The recurrence of T1D in pancreas transplant recipients was initially reported by Dr David Sutherland in cases where patients received living-related pancreas grafts from twins or HLA-identical siblings and, due to HLA identity, received little to no immunosuppression (209). Observations of relapse of autoimmunity as assessed by autoantibodies and occasionally T cells have also been reported following allogeneic pancreas transplant under immune suppression (210–216).

Although the cases of islet transplants are far fewer than pancreas transplants, there is good evidence to indicate that transplantation of isolated allogeneic islets can cause relapse of autoimmunity in a small but significant portion of patients (199, 217–221). Occasional patients had dramatic rises in islet autoantibodies from around day 5 after transplant that occurred without any sign of allo-immunity (222). Weaker immunosuppression regimes such as MMF plus 1,25 (OH)2 Vit D3 were more frequently associated with a sharp immediate risk in autoantibodies with and without allo-reactivity. Others showed that T cell responses to islet autoantigens are often increased after islet transplants (223, 224). Although associations with reduced graft function have been reported (221), it is not fully proven that the relapsing autoimmune response post islet transplantation equals autoimmune mediated destruction of islet grafts. It’s worth mentioning that during immunosuppression and the use of immunodepleting agents, lymphopenia can significantly contribute to the expansion of memory autoreactive T cells (225). This expansion is primarily driven by homeostatic proliferation, which is strongly influenced by the IL-7/IL-7 receptor axis (226). The existence of homeostatic proliferation among effector T cells, including clones of autoreactive T cells, in individuals undergoing islet transplantation (227). Furthermore, it has been demonstrated in various cases, such as the transfer of T1D between siblings after bone marrow transplantation (228) and the development of T1D following islet auto transplantation within the first year after pancreatectomy (229, 230), that autoimmune reactions alone can lead to the destruction of newly transplanted beta cells. With the advent of innovative techniques for producing β cells from readily available pluripotent stem cell sources, concerns pertaining to allorecognition, and HLA matching can be effectively addressed. One approach involves the establishment of master cell banks comprising stem cell-derived β cells that match the major histocompatibility complex (MHC) class I and II alleles commonly found in individuals with T1D. Alternatively, thanks to the capabilities offered by CRISPR-Cas9 gene editing, it becomes feasible to create “stealth” β cells that can evade immune recognition by disabling endogenous HLA molecules. Additionally, in preparation for the potential resurgence of autoimmune responses, an effective strategy may require the prior reduction of autoreactive memory, along with the conditioning of repopulating lymphocytes to promote enduring immune tolerance. These forthcoming opportunities will be discussed in more detail above.

## 3 Unanswered questions and persistent knowledge gaps in the immunological challenge of Allo Beta Cell transplantation

Several aspects of the immunological challenge associated with Allo Beta Cell Transplantation continue to elude our complete understanding. These gaps in knowledge raise important questions about the precise mechanisms and factors influencing the success and longevity of beta cell replacement therapies. Addressing these gaps is crucial for advancing our comprehension of the immune response to transplanted beta cells and devising more effective strategies to ensure the sustained function and survival of these cells in individuals with conditions like T1D.

### 3.1 Lack of evidence on immunosuppression treatment

**Figure 1:**
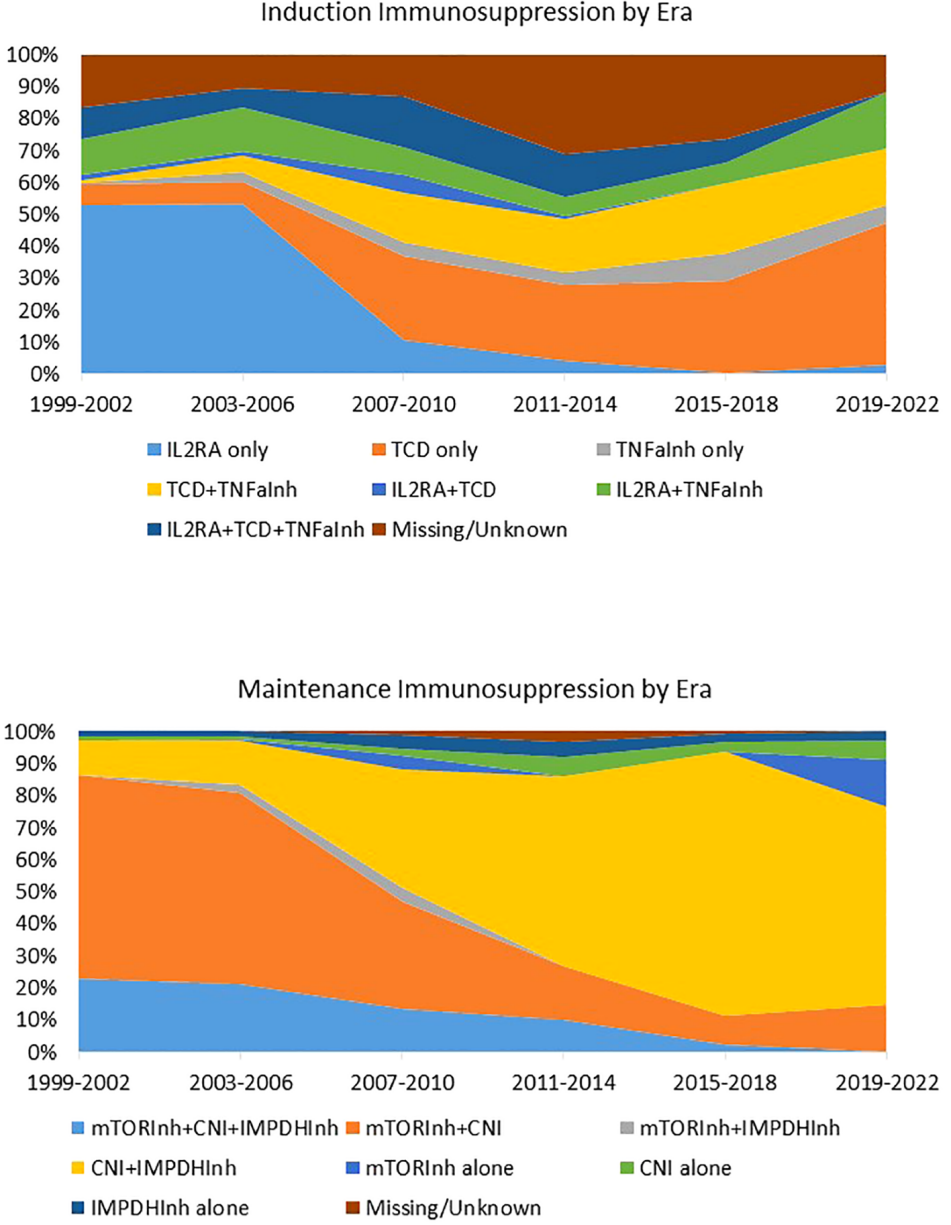
Induction and maintenance immunosuppression in islet transplantation by era. Immunosuppression regimen of 1,108 individuals with T1D who received Islet Transplant Alone (n = 992) or Islet after kidney (n = 186) between 1999 and 2022 and were followed by the CITR. Data source: Collaborative Islet Transplant Registry Coordinating Centre: Eleventh allograft report 2022. TCD, T cell depleting agents; Inh, inhibitor; CNI, calcineurin Inhibitor; IMPDH, Inosine-5′-monophosphate dehydrogenase; IL1RA, IL1 receptor antagonist. Reproduced from “Caldara R, Tomajer V, Piemonti L. Enhancing Beta Cell Replacement Therapies: Exploring Calcineurin Inhibitor-Sparing Immunosuppressive Regimens. Transpl Int. 2023 Jun 8;36:11565” with permission from the authors.

In contrast to most solid organ transplantations, there is currently no available guidance or formal consensus on the optimal or standard immunosuppressive strategy for Allo Beta Cell Transplantation. This critical gap has led to a significant evolution in immunosuppression approaches over the years, all without the benefit of evidence-based practices (as illustrated in **Figure 1**). Numerous studies, often conducted on limited patient cohorts, have proposed a variety of immunosuppressive agent combinations (59, 231–233). These encompass agents that deplete T and B cells (such as alemtuzumab, teplizumab, antithymocyte/lymphocyte globulin, rituximab), inhibitors of T-cell activation (like IL2R antagonists daclizumab and basiliximab), replication inhibitors (including azathioprine and mycophenolate mofetil/mycophenolic acid), mTor inhibitors (such as sirolimus and everolimus), lymphocyte tracking inhibitors (like EFA), desensitizing agents (such as intravenous immunoglobulin), co-stimulation inhibitors (including monoclonal antiCD28 belatacept/abatacept), CNIs (cyclosporine and tacrolimus), and anti-inflammatory agents (including corticosteroids, IL1 receptor antagonist, and TNF-alpha inhibitors). It is crucial to emphasize that most of these studies have been observational, consisting of retrospective or prospective single-center single-arm studies. Remarkably, there is only one recently reported randomized controlled trial study that has emerged as an exception, focusing on CXCR1/2 inhibitors (62). Many immunosuppressive drugs used in Allo Beta Cell Transplantation are designed to inhibit specific pathways of alloantigen specific T cell activation, but they ignore the memory autoimmune response, and they were quite ineffective in controlling the IL-7 mediated homeostatic proliferation.

### 3.2 Lack of reliable method to assess rejection and treatment protocol

Unlike most solid organ transplantations, there is currently no consensus on how to diagnose Allo Beta Cell Transplantation rejection (234). This challenge arises because the traditional gold standard for diagnosing rejection involves tissue biopsy (235). While a whole organ pancreas transplant biopsy can yield valuable insights, particularly for potentially reversible causes of dysfunction, technical challenges limit its routine application. Given that isolated islet transplantation is accomplished by infusing pancreatic islets into the portal circulation, where they disperse throughout the liver, accessing the islet graft for regular biopsies or surveillance becomes unfeasible. Hence, there is a pressing need for standardized clinical diagnostic criteria that can effectively identify ongoing islet allograft rejection. Moreover, there are currently no established treatment protocols in place for Allo Beta Cell Transplantation rejection, which may be related to a paucity of data on diagnostic criteria (236–238). While high-dose steroid therapy is a potential avenue for halting ongoing cellular rejection (234), it’s crucial to note that this therapy itself is associated with a possible decline in the functional performance of islet grafts. Furthermore, there have been suggestions for addressing humoral rejection through the utilization of rituximab and IV immunoglobulin therapy, though these recommendations are primarily based on single case reports (237).

### 3.3 Lack of studies to assess the efficacy of immunologic and metabolic testing to detect early graft dysfunction after Allo Beta Cell transplantation

Undoubtedly, the field of Allo Beta Cell Transplantation faces a conspicuous absence of sensitive, non-invasive serial assays for the early detection of rejection or autoimmune recurrence and the ongoing loss of beta-cell functional mass (239). While a consensus has recently been established for defining clinically successful graft functional outcomes in beta-cell replacement therapies (240), there is still a notable absence of standardized and systematic immunologic and metabolic monitoring protocols following Allo Beta Cell Transplantation. Parameters such as body weight, fasting glucose levels, fasting and random C-peptide concentrations, fasting insulin levels, HbA1c measurements, oral glucose tolerance tests (OGTT), mixed meal tolerance tests (MMTT), insulin clamp studies, continuous glucose monitoring (CGM), assessments for anti-donor human leukocyte antigen antibodies (specifically donor-specific antibodies, DSA), and the monitoring of autoantibodies have been commonly employed by experienced programs worldwide, albeit with varying time schedules and indications (either protocol-driven or initiated “for cause”). However, their effectiveness in detecting early graft dysfunction, particularly at a stage when timely clinical intervention can forestall further deterioration and preserve allograft function, remains unproven (239). Additionally, there remains an ongoing debate regarding the predictive role of certain immunological parameters in graft failure. In the broader context of solid organ transplantation, donor-specific antibodies (DSA) are recognized as the primary culprits behind graft failure. Preexisting DSA serves as a relative contraindication to transplantation, and the emergence of *de novo* DSA plays a pivotal role in antibody-mediated rejection, leading to microvascular inflammation and associated with unfavorable outcomes. In the context of islet transplantation, there have been descriptions of the potential adverse effects of *de novo* DSA (199, 241, 242), although not all studies have confirmed this association (243–245). Furthermore, while DSA and assays for islet autoantigen antibodies are well-established and reproducible worldwide, the consistency of other assays and biomarkers remains variable. Emerging assays and platforms designed to assess cellular responses to auto/alloantigens and those focused on donor-derived cell-free deoxyribonucleic acid (dd-cfDNA) are examples of these less established tools that have not yet achieved universal consistency and acceptance.

### 3.4 Lack of validate biomarker for beta cell death

The current absence of real-time biomarkers for monitoring beta cell death presents a significant challenge in Allo Beta Cell Transplantation. Detecting the loss of islet beta cells after transplantation relies on assessing glycemic control, the need for external insulin supplementation, and measuring insulin secretion, often by evaluating C-peptide levels. The introduction of more sensitive indicators has the potential to facilitate interventions that can prevent clinically significant islet graft loss. Such indicators could be particularly valuable for guiding immune monitoring of humoral and cellular alloimmune and autoimmune markers or for interpreting the potential significance of newly detected alloantigen or autoantigen reactivity in a transplanted islet beta cell graft before any functional deterioration becomes apparent. Various methods have been employed to identify impaired islets in the bloodstream shortly after intraportal infusion, as up to 25% of the transplanted islet mass may be lost. These methods encompass the examination of insulin mRNA (246, 247), glutamic acid decarboxylase-65 (GAD65) (248), miRNA375 (249, 250), and unmethylated insulin DNA (251, 252). These markers have been observed to elevate within 24 hours after islet transplantation, with some being associated with worse islet graft functional outcomes and modulation by anti-inflammatory therapy during the first week post-transplantation. This suggests their potential utility for both predicting early engraftment and assessing interventions aimed at enhancing islet survival during the engraftment period. However, more sensitive, and reproducible assays are needed to detect subtler episodes of cell death that may provide insights into graft rejection or recurrent diabetes.

### 3.5 Lack of beta cell imaging

Over the past two decades, research in the field of non-invasive beta-cell imaging and beta-cell mass evaluation has witnessed progress (253, 254). This includes the identification of target molecules for imaging probes, the development of chemically modified probes labelled with suitable radioisotopes, and the establishment of analytical methods for signal interpretation through single-photon emission computed tomography and positron emission tomography. Notably, derivatives of exendin-4 designed for imaging show promise as candidates for non-invasive beta-cell mass assessments. However, the non-invasive evaluation of beta-cell mass remains elusive, and practical *in vivo* and clinical techniques for β-cell-specific imaging are yet to be established (253).

## 4 Emerging directions and game-changing strategies in addressing the immunological challenge of Allo Beta Cell transplantation

The field of Allo Beta Cell Transplantation is witnessing a transformative shift with the emergence of innovative strategies aimed at overcoming the immunological challenges inherent to the procedure. These groundbreaking approaches have the potential to revolutionize the field and significantly enhance the success and sustainability of beta cell replacement therapies.

### 4.1 Exploring alternatives to conventional immunosuppressive regimens

Allo Beta Cell Transplantation holds great promise as a therapeutic avenue for individuals grappling with T1D, as it offers the potential for achieving insulin independence and markedly improved glycemic control. Nevertheless, the success of this approach is inextricably linked to the adept management of immune responses, a vital factor for thwarting graft rejection and addressing the autoimmune components of the condition. In recent times, the field has witnessed the ascent of various novel approaches within immunosuppression strategies, all geared towards elevating the overall success and accessibility.

#### 4.1.1 Exploring calcineurin inhibitor and depleting agent sparing immunosuppression

Traditional immunosuppressive protocols, while effective in preventing rejection of transplanted organs, often carry the burden of long-term side effects and may not offer comprehensive protection against both alloimmune and autoimmune responses. These customary treatment regimens typically encompass medications like Calcineurin Inhibitors (CNIs), such as cyclosporine and tacrolimus, among others. While these drugs effectively suppress the immune system, achieving their intended objectives, they are not devoid of drawbacks. Prolonged use of CNIs can give rise to complications and their known beta cell (255) and renal toxicities (256) limit their efficacy for pancreas and islet transplantation. These adverse effects can impact a patient’s overall well-being and quality of life. Furthermore, CNIs’ involvement in the nuclear factor of activated T cells (NFAT) signaling pathway, which is pivotal for the differentiation, maintenance, and suppressive capabilities of Tregs, can have significant repercussions (257). This involvement may hinder the establishment of immune tolerance and impede the effectiveness of potential adoptive therapies employing tolerogenic donor specific Tregs (as discussed below). Additionally, it’s important to note that CNIs have no impact on T cell expansion during homeostatic proliferation since they effectively block the IL-2 pathway but are comparatively ineffective in regulating IL-7-mediated homeostatic proliferation (225). Given these considerations, there is a pressing need for research to investigate the safety and feasibility of immunosuppressive regimens that reduce the reliance on CNIs. Equally troublesome is the use of depleting agents like ATG and alemtuzumab (anti-CD52) for induction. These agents can significantly influence the severity of lymphocyte depletion and potentially affect the rate of cell cycling during reconstitution. Lymphocyte depletion therapies with alemtuzumab or ATG can lead to the expansion of alloreactive and autoreactive T cells, in some cases exceeding pretransplant levels (258, 259). Alemtuzumab treatment has been demonstrated to preferentially expand effector-memory T cells in renal transplant recipients, while induction with ATG expands both effector-memory and central-memory T cell subsets. Furthermore, these agents can pose challenges for the development of adoptive therapy with tolerogenic T regulatory cells, which could be equally recognized and depleted, like conventional T cells. Notably, even non-depleting anti-CD25 monoclonal antibodies could present issues in terms of homeostatic proliferation, as they were specifically designed to prevent the formation of the high-affinity IL-2R complex and block IL-2 signaling. The limited availability of the common gamma chain shared by IL-2 and IL-7 receptors represents a constraint on cytokine signaling. When the formation of the IL-2 receptor is inhibited by non-depleting anti-CD25 monoclonal antibodies, more common gamma chain becomes available for complexing with IL-7R alpha, resulting in increased T cell sensitivity to IL-7 and favoring homeostatic proliferation (260). Considering these considerations, conducting research on immunosuppressive regimens that minimize the use of CNIs and avoid induction with depleting agents will significantly advance beta cell replacement therapies. Some prior small-scale clinical experiences have already demonstrated the value and feasibility of these approaches. Feasibility, safety, and efficacy of CNIs-free and anti-IL-2Ra-free treatments for islet transplantation, which also exclude anti-thymocyte globulin induction during second or third infusions, have been successfully demonstrated (261). More recently, reports have surfaced of 40% insulin independence at 10 years following a single islet infusion with CNI-sparing immunosuppressive regimens, including either belatacept (BELA) or efalizumab (EFA). These regimens have showcased remarkable cases of operational tolerance and substantial expansions of Tregs following islet transplantation (262). Furthermore, the identification of biological and pharmacological controllers of the IL-7/IL-7R axis, which hold promise for potential clinical applications, could be pertinent to the development of advanced immunosuppressive protocols for Allo Beta Cell Transplantation (226).

#### 4.1.2 Rethinking induction therapy and exploring recipient preconditioning

Induction therapy has proven to be an effective strategy for achieving low rates of acute rejection in most allograft situations (263). he necessity for induction immunosuppression arises from the heightened immunogenicity of the allograft during the immediate post-transplant period. Specifically, this vulnerability is attributed to the combined factors of a high frequency of donor-specific T-cell precursors present in most recipients and the activation of the innate immune system during organ transplantation (264). This established approach was developed in a clinical context where organ availability is unpredictable, and the time between organ donation and transplantation falls within a matter of hours. This limitation made it impractical to consider recipient pretreatment longer than 1-2 days or any donor-specific preconditioning strategies. As a result, induction therapy primarily aimed at achieving short-term profound immunosuppression without a focus on long-term sustainability. However, this paradigm could be revolutionized in the realm of Allo Beta Cell Transplantation. The availability of insulin-producing cells derived from replenishable sources like stem cells introduces the possibility of scheduled transplants with known and defined timeframes, along with prior characterization of the donor’s MHC profile. This scenario opens new avenues in induction immunosuppression, encompassing approaches such as costimulation-based therapy, mixed chimerism, and adoptive cellular transfer. These innovative strategies aim to restore immunological balance in the context of organ transplantation rather than relying on non-specific immunosuppression. Some experiences have already demonstrated the value and feasibility of these approaches. Some experiences have already demonstrated the value and feasibility of these approaches. For instance, administering apoptotic donor leukocytes around the time of transplant, in conjunction with short-term immunotherapy involving antagonistic anti-CD40 antibody 2C10R4, rapamycin, soluble tumor necrosis factor receptor, and anti-interleukin 6 receptor antibody, has been shown to induce long-term (≥1 year) tolerance to islet allografts in rhesus macaques (265). Similarly, recipient preconditioning with GLP-1 agonists or rapamycin has been proposed as an effective strategy for enhancing graft function in both preclinical and clinical models (266–268). Indeed, this shift in perspective toward induction and recipient preconditioning invites us to reconsider conventional approaches and fosters the exploration of innovative strategies to enhance the field of Allo Beta Cell Transplantation.

#### 4.1.3 Targeting autoimmunity recurrence and beta cell survival

The diabetes community has long anticipated the use of immunosuppressive treatments in individuals with recent-onset T1D and those at risk of developing the disease (269). Currently, aside from the FDA-approved anti-CD3 antibody teplizumab (270), no such treatment is in clinical use. However, recent publications suggest promising strategies in this regard. For instance, low-dose ATG has demonstrated its effectiveness in maintaining C-peptide levels compared to a placebo (271). Teplizumab, in trials involving individuals at high risk of T1D, doubled the time to disease onset compared to a placebo (270). On the other hand, anti-CD3 Otelixizumab failed in its phase III trial. Alefacept, which targets CD2 primarily expressed on CD4+ and CD8+ effector memory T cells, has been tested in recent-onset T1D and displayed C-peptide preservation along with reduced use of exogenous insulin compared to a placebo group (272, 273). Other trials have explored various approaches to combat islet autoimmunity. These include CTLA-4Ig (abatacept) (274), anti-CD20 therapy (rituximab) (275), anti-TNF-α therapies (recombinant TNF-α receptor-IgG fusion protein etanercept and IgG1-κ monoclonal anti-TNF-α antibody golimumab) (276, 277), anti-CD40 therapy (Iscalimab) (278), low-dose IL-2 (279), IL-1 blocker (Anakirna) (280), combination immunomodulatory and beta-cell therapy like anti-IL-21 antibody and liraglutide (281), and Tyrosine Kinase Inhibitors (Imatinib mesylate) (282). While these immunosuppressive regimens have been evaluated to varying degrees of success in recent-onset T1D, exploring these candidates, or future ones, for their ability to attenuate autoimmune responses in beta-cell graft recipients offers new avenues for immune suppression.

### 4.2 CAR T reg and TCR T reg

The donor beta cells express allogeneic major and minor histocompatibility antigens, traditionally targeted by the host immune response in the setting of organ transplantation. Moreover, the donor beta cells also express a full complement of antigens associated with islet autoimmunity. Of these, glutamic acid decarboxylase 65 (GAD65), insulinoma-associated protein 2, zinc transporter 8 (ZnT8) and (pro)insulin appear to be highly antigenic in humans both for T cells and B cells (166). Beta-cell replacement into a subject with pre-existing autoimmunity is essentially an immunological challenge where conceptually similar immune responses—transplant rejection and tissue-specific autoimmunity—coexist, but with the potential for reactivation of autoreactive memory T and B cells posing an additional set of therapeutic obstacles. Adoptive cell therapy using CD4+CD25+FOXP3+ Tregs, a naturally suppressive immune subset, is a promising approach to achieving localized and specific immune suppression in the site of transplant (283). However, clinical trials testing administration of polyclonal Tregs in recent-onset T1D have observed limited efficacy despite an excellent safety profile (284, 285). Similarly, administration of autologous Tregs together with intraportal allogeneic islet transplantation yielded no severe negative effects (286). These clinical trials have been fundamental to identify barriers to an effective Treg therapy. First, the use of polyclonal Treg for adoptive cell therapy relies on the assumption on the natural existence of rare, disease relevant TCRs in the adoptively transferred Treg population. However, different studies in NOD mice reported that therapy using antigen specific Tregs is far more effective than the one using polyclonal Treg. Notably, one study found that transfer of 2 million antigen-specific (BDC2.5 TCR transgenic) Tregs controlled the rejection of a syngeneic islet transplant in NOD mice, whereas 5 million polyclonal Tregs displayed no effect (287). The recent emergence of advanced gene editing techniques has opened new avenues to engineer Tregs with selected antigen specificity (288). These include the generation of Treg bearing a chimeric antigen receptor (CAR-Treg) as well as T cells bearing a transgenic T cell receptor (TCRtg-Treg) with a selected antigen specificity. CAR are composed by an extracellular antigen-binding domain, usually a single-chain variable fragment (scFv) derived from the variable regions of an antibody linked via hinge and transmembrane domains to an intracellular signaling domain (289). CAR do not need to be MHC-restricted, allowing the use of the same CAR on virtually all subjects independently from their HLAs. Moreover, modern CAR are designed as modular systems in which the signaling pathway activated by antigen recognition can be adapted to the desired effect (290). A notable disadvantage of CARs is the requirement for cell-surface bound target antigen whose expression ideally must be confined to beta-cells. The difficulties in finding a good target antigen on beta-cells has considerably limited the used of CAR-Treg to control autoimmunity in type I diabetes. However, transplanted allogenic beta-cells express mismatched HLA molecules that can be easily targeted by CAR. Human CAR Tregs that target the commonly mismatched HLA-A2 molecules are currently being tested clinically in kidney (NCT04817774) and liver transplantation (NCT052334190). TCRtg-Treg are easier to develop in the context of beta-cell autoimmunity. Indeed, a number of TCRs specific for epitopes of GAD65, preproinsulin, IGRP as well as neo-epitopes have already been identified from patients with type I diabetes (291, 292). While MHC restriction can represent a limitation, the use of target peptides associated to commonly expressed HLAs, such as HLA-A2 or T1D risk associated class II haplotypes, potentially allows to treat a significant proportion of subjects with relatively few different TCRtg-Treg. TCRtg-Treg also requires additional gene editing to be fully functional. Suppression of the endogenous TCR is needed to improve expression of the transgenic TCR but also to avoid mispairing of the endogenous and transgenic TCR alpha and beta chains, potentially impairing beta-cell antigen-specificity and increasing the risk of off-target antigen recognition (293). It has also to be determined whether peptide/HLA class I restricted TCR can efficiently recognize the antigen when transduced into CD4+ Treg and whether transgenic expression of CD8 can improve antigen recognition. With several important issues yet to be determined, Abata Therapeutics have recently announced the development of a beta-cell specific TCRtg-Treg product (ABA-201) that will be clinically tested in 2025. A second important limitation of adoptive Treg therapy is the survival and persistence of Treg transferred in patients that may impact the therapeutic effect. Bluestone et al. (NTC01210664) observed a rapid decline in the number of circulating Treg following adoptive transfer into patients with T1D. Specifically, once infused into patients, the ex vivo expanded Treg population exhibits a biphasic exponential decay kinetic, characterized by a short-lived subset (75-90%) with a half-life of a few days to weeks, and a long-lived subset (10-25%) detectable up to one year post-infusion (284). Notably, although the expanded Treg initially display a CCR7+CD45RO+CD45RA- central-memory phenotype, the subset that survives longer in patients exhibits a CCR7+CD45RA+CD45RO-/+ phenotype, resembling that of conventional naïve or memory stem T cells. Addressing the issue of Treg survival, a second trial involving adoptive transfer of polyclonal Treg cells along with exogenous administration of low doses of recombinant human IL-2 was conducted (279) (NCT02772679). Addition of IL-2 did not improve the survival of adoptively transferred Treg but was associated with increase endogenous Treg numbers and expansion of inflammatory NK and CD8+GMZB+ T cells. Several other strategies to promote Treg survival in patients are under intensive studies. Notably, synthetic orthogonal receptor-ligand pair has been generated. In this approach T cells are transduced with an orthogonal IL-2 receptor that can only be activated by an exogenously administered synthetic ligand (294). An alternative approach is to transduce Treg with a membrane-bound form of IL-2, in which IL-2 is tethered to the membrane by a short linker that only allows cis-interactions between IL-2 and its receptors on the same cell (295). As Treg need to be expanded *in vitro* to achieve a number sufficient to display therapeutic effectiveness, modification to the expansion protocols to improve T cell survival are under consideration. Tregs are traditionally expanded using anti CD3/CD38 microbeads in combination with high doses of interleukin 2 (IL-2) (296). Despite low expression of the IL-7Ralpha (CD127) human naïve Treg have been shown to respond to and proliferate in response to IL-7 *in vitro* (297). Furthermore, in conditions of Treg depletion, IL-7 contributes to Treg compartment reconstitution in patients treated with the anti-CD25monoclonal antibody basiliximab (226). A novel protocol of expansion of Treg using a combination of IL-2 and IL-7 was shown to improve the survival of Treg in the NSG mouse model (298). As transplanted beta-cells can be targeted by allo-reactive and auto-reactive T cells, adoptive Treg therapy represent an opportunity to keep T cell responses in check. While the clinical testing, especially in T1D, has provided clear results in terms of safety but also highlighted several critical issues that need to be addressed, and effective Treg therapy can be available in the coming years. Specifically in the transplantation setting an additional effort is required to determine T cell survival, persistence, and therapeutic effectiveness when Treg therapy is administered in combination with immune-suppressive drugs.

### 4.3 Stem stealth cells

Stem cell technology has ushered in a new era in β cell generation for transplantation. “Stem stealth cells” represent a novel concept where stem cells are genetically modified to evade immune recognition. The first and one of the most successful strategies to reduce immunogenicity is the abrogation of the Beta-2 microglobulin (B2M) gene, which encodes a common subunit of HLA class I molecules. Knocking out B2M results in HLA class I-negative iPSCs, which can function as universal donors for the transplantation of cells that do not express HLA class I (299). Several methods have been developed to disrupt the B2M gene in ESCs and iPSCs. For example, one study used Cre-recombinase to ablate two adeno-associated virus (AAV)-inserted cassettes into exon one of the B2M gene. This method successfully silenced B2M expression and resulted in reduced allogeneic responses of T cells (299). A second study employed CRISPR/Cas9 technology to target exons 2 and 3 of the B2M gene, replacing them with other genetic cassettes (300). These cells were resistant to interferon-γ stimulation and alloreactive CD8+ T cells, indicating that they do not express cell surface human leukocyte antigen (HLA) molecules. Additionally, these B2M-/- hESCs do not have any off-target integration or cleavage events, lack stable B2M mRNA, have a normal karyotype, and maintain their self-renewal capacity, genomic stability, and pluripotency. To validate the potential of these strategies, preclinical studies have demonstrated the feasibility of B2M-knockout iPSCs in various transplantation models. B2M-null iPSC-derived cells, such as neurons, cardiomyocytes, and retinal pigment epithelial cells, have been successfully transplanted into animal models, with extended survival and functional integration compared to their HLA-mismatched counterparts (301–303). These promising findings highlight the potential of B2M-knockout cells as universal donors for cell-based therapies. The limit of this approach is that the B2M-null cells are protected from CD8+ T cell responses but become more susceptible to NK cell-mediated destruction (300). To address this issue, new strategies were developed to express specific ligands on the cell surface that interact with inhibitory receptors on NK cells, rendering them less cytotoxic. One such ligand is human leukocyte antigen-E (HLA-E), which interacts with inhibitory receptors such as NKG2A/CD94 on NK cells, leading to their inhibition (304, 305). Expressing HLA-E on the surface of iPSC-derived cells has been shown to protect them from NK cell-mediated lysis (301, 306). In addition to HLA-E, HLA-G, another member of the HLA family with immunosuppressive properties (307) has also been explored (308, 309). Recently, innovative approaches which involve editing iPSC to remove NK-activating ligands, such as CD155 and B7-H3, have been proposed. These ligands, when expressed on the cell surface, can trigger NK cell cytotoxicity (310). By eliminating these ligands, iPSC-derived cells resulted more resistant to NK cell-mediated killing (311). This work also proved that the capacity to differentiate into β cells was not impaired in gene edited iPSC and that iPSC-derived pancreatic cells were able to survive *in vivo* after transplantation in mice, while unedited cells were eliminated by NK cells.

Another component of the immune system involved in rejection is CD4+ T cell, which helps to coordinate the immune response by stimulating other immune cells, such as macrophages, B lymphocytes, and CD8 T lymphocytes. HLA II defected hESC were generated via deleting CIITA, a master regulator of constitutive and IFN-γ inducible expression of HLA II genes. CIITA-/- ESC can differentiate into tissue cells with non-HLA II expression and escape the attack of receptors’ CD4+ T cells (302, 312). These strategies and the possibility to combine them hold great promise in enhancing the immune evasion capabilities of transplanted cells.

In addition to approaches that directly aim to escape cytotoxic cell recognition, the induction of tolerogenic genes within transplanted cells to create a more immune-tolerant microenvironment was explored. Several genes have been investigated for their potential to suppress immune responses and promote graft acceptance:

- PD-L1 (Programmed Death-Ligand 1): PD-L1 is an immune checkpoint protein that interacts with the PD-1 receptor on T cells, leading to T cell exhaustion and immune tolerance (313). Studies have shown that overexpressing PD-L1 in iPSC-derived cells can mitigate T cell responses and enhance graft survival (314).
- CTLA4-Ig: Cytotoxic T lymphocyte-associated protein 4-immunoglobulin (CTLA4-Ig) is a fusion protein that binds to CD80 and CD86 on antigen-presenting cells, preventing their interaction with CD28 on T cells. This blockade inhibits T cell activation and promotes immune tolerance (315). Incorporating CTLA4-Ig expression into transplanted cells has demonstrated success in prolonging graft survival (314).
- CD47: CD47 is a cell surface protein that acts as a “don’t eat me” signal by binding to the signal-regulatory protein alpha (SIRPα) on phagocytic cells, inhibiting their engulfment of the CD47-expressing cell (316). Enhancing CD47 expression on iPSC-derived cells has been shown to reduce their susceptibility to phagocytic clearance (317, 318).
- IDO (Indoleamine 2,3-Dioxygenase): IDO is an enzyme that plays a role in immunosuppression by degrading tryptophan, an essential amino acid for T cell proliferation (319). By overexpressing IDO in islet cells, researchers have aimed to create a tolerogenic microenvironment that inhibits T cell responses and promotes graft survival (320).

These gene-based strategies aim to create a microenvironment within transplanted cells that is conducive to immune tolerance, thereby improving the long-term survival of grafts. These approaches collectively represent a growing toolbox of strategies to improve the success of islet transplantation without the need for extensive immunosuppressive regimens. To demonstrate this, very recently, gene editing techniques that combine targeting of HLA class I and II and the immunomodulatory gene CD47 were tested in human donor islets, modifying them to become hypoimmune (HIP). It was demonstrated that these human HIP islets could survive, engraft, and improve diabetes in allogeneic, diabetic humanized mice. Furthermore, the HIP islet cells exhibited the ability to evade autoimmune destruction in autologous, diabetic humanized autoimmune mice (318). The same approach of HLA class I and II depletion and CD47 overexpression (B2M-/-CIITA-/-CD47+) was used in rhesus macaque HIP stem cells, which were transplanted into four allogeneic rhesus macaques. The HIP cells demonstrated unrestricted survival for 16 weeks in fully immunocompetent allogeneic recipients and differentiated into various lineages, whereas allogeneic wild-type cells were strongly rejected. Additionally, human HIP cells were differentiated into pancreatic islets and shown to survive in immunocompetent, allogeneic diabetic humanized mice for 4 weeks, effectively ameliorating diabetes. Edited primary rhesus macaque islets with HIP modifications were able to survive for 40 weeks in an allogeneic rhesus macaque recipient without the need for immunosuppression, whereas unedited islets were rapidly rejected (321).

This last evidence supports the strategy to use gene engineering to make stem cell-derived and isolated islet transplants less visible to the host immune system, thereby increasing the likelihood of successful transplantation and reducing the dependence on long-term immunosuppressive therapy. From the abrogation of the B2M and CIITA genes to the modulation of NK ligands, these innovative ways could protect transplanted cells from immune responses (311). Moreover, the induction of tolerogenic genes like PDL-1 and CD47 and the engineering of immune-evasive islets have shown promise in creating a more immunologically tolerant microenvironment within the transplanted cells (322).

Despite these remarkable advancements, challenges remain on the path to clinical implementation. The long-term safety of these immune-evasive strategies need to be rigorously evaluated. In fact, hypoimmunogenic cells may raise potential safety concerns associated with long-term immune surveillance and malignancy risk: a hypoimmunogenic transplant may evade immediate immune responses, but the long-term ability of the recipient’s immune system to recognize and respond to potential threats, such as malignancies or chronic infections, may be compromised. Besides, a suppressed immune system may be less effective in preventing the growth and spread of tumor cells. One possible strategy to increase the safety of hypoimmunogenic cell would be to equip the cell with a safety switch, able to induce cell suicide in case of abnormal cell proliferation and tumorigenesis (323, 324).

Finally, the safety of genetic manipulation must be considered, and safety improvements achieved by the thoughtful design of nucleases and repair templates, the analysis of off-target editing, and the careful utilization of viral vectors (325–327). The development of new generations of gene editing tools will hopefully bring to improved targeting of specific sequences while minimizing the risk of unintended outcomes.

In conclusion, if combination of gene editing immunological targets will prove effective and safety requirements will be satisfied, stem stealth cells have the chance of serving as a replenishable and customizable source of βcells for transplantation, mitigating the risks associated with immune rejection.

### 4.4 Tissue engineering and encapsulation

Tissue engineering and encapsulation technologies have made remarkable progress in creating protective microenvironments for transplanted beta cells, reshaping the landscape of diabetes treatment (328, 329). Micro and macro-encapsulation devices function as essential shields, safeguarding cells from immune attacks while facilitating the crucial exchange of oxygen and nutrients. In this scenario, a valuable lesson has been gleaned from the clinical experience of Viacyte, emphasizing the need for a swift transition from a closed to an open device to facilitate vascular scaffold connection (117). This underscores the importance of considering the mandatory requirements of beta cells in terms of nutrient supply and vascular integration in tissue engineering for beta cell replacement. Concurrently, ongoing initiatives in tissue engineering are focused on the development of bioengineered scaffolds that closely mimic the natural pancreatic microenvironment, thereby enhancing the survival and function of transplanted cells (329). These innovative strategies not only shield beta cells from immune threats but also facilitate seamless integration and sustained functionality within the host environment (328). These technologies, ranging from organ engineering (330) to cutting-edge 3D-bioprinting (331), play a pivotal role in modulating the endocrine niche before transplantation. This modulation is achieved by intricately integrating various cell components within an extracellular matrix (ECM) framework. Dedicated bioreactors enable the repopulation of these constructs with different target cells, matured to acquire new scaffold functions. For example, the repurposing of organ strategies has transformed decellularized rat lungs into structures repopulated with pancreatic islet and endothelial cells, generating a vascularized endocrine pancreas (332, 333). These new devices exhibit matured vascularized endocrine structures, resembling the pancreatic endocrine niche prior the implantation, displaying both ex vivo and *in vivo* functionality. This versatility in cell selection may allow, in the future, for the design of immunomodulation strategies during the engineering process, reducing device immunogenicity and enabling the delivery of immune-modulatory compounds.

In the first scenario, a viable solution involves selecting autologous endocrine niche cells to create an open vascularized device, significantly reducing immunogenicity from a transplantation perspective. While autologous cells sourced from stem cells are prone to autoimmune responses, recent gene editing advancements have generated cell sources from stem cells or even human islet or pig donors, evading both auto and allo or xeno-immune responses (318, 334, 335). This progress empowers the assembly of innovative open devices, ensuring complete structural integration and genetically engineered immune protection. Although these strategies look promising, they are still evaluated in advanced preclinical stage and further tests will be required to move in clinical arena. In the second scenario, immunomodulatory compounds are delivered within the device and locally released at the transplant site, minimizing compound toxicity, and enhancing local drug efficacy. In this context hydrogels are widely used as cell encapsulation technology, as their mechanical properties, along with the high hydration degree, mimic soft tissues. They can be synthesized in the micro and macro scale, which typically imposes a volume increase that prevents intrahepatic infusion. They have been largely tested within beta cells and have been demonstrated to safely integrate with the recipient allowing vascularization *in vivo* (336, 337). Multiple engineered scaffolds have been developed to deliver immunomodulatory compounds or apoptosis modulators in hydrogel form, dampening or halting the immune-mediated graft response (338, 339). These attempts reported, in preclinical setting promising result in protecting beta cells from recipient immune attack. Additional experimental are on-going to observe long term function of this devices and their efficacy in protecting the graft based on local immunomodulatory compounds with pancreatic islet or stem cell derived beta-cells form immune recognition (340). Alternatively, advanced macro-engineering devices are pre-implanted to foster vascular integration. Subsequently, these devices can be loaded with pancreatic islets and immune-suppressant drugs, shielding engrafted pancreatic islets from inflammatory and immunological reactions. Recent data, in both rodent and human primate model, have demonstrated the effectiveness of this technology in protecting engrafted cells and constraining the immune reaction against the graft in the presence of a reduced early engraftment due to the time of connection of the seeded islet within the new generated vascular network.

In the evolution of tissue engineering approaches for beta cell replacement, a critical role has also the selection of the implantation site that can affect, from oxygen, nutrient supply and immunological activity, the outcome, and the translatability of the results. Despite the agreement that an extrahepatic site for islet transplantation is needed, non a common consensus have been released on the best alternative site for device implantation. The most used is the subcutaneous space considering its exposure and flexibility in case of device retrieval. In this direction a recent study has introduced a cutting-edge computational platform. This platform aims to explore the therapeutic potential of programmable bioartificial pancreas devices (341). The study employed sophisticated software that considered factors such as cell load and site-specific oxygen levels. This analysis allowed for precise adjustments in terms of cell loading and oxygen supply within the device, marking a significant stride in the field of tissue engineering for diabetes treatment. Looking ahead, artificial intelligence (AI) tools are poised to play a pivotal role in advancing beta cell replacement technology (342). By leveraging AI, researchers can amalgamate intricate details such as scaffold designs, transplantation site characteristics (including vascularization and immunoreactivity), and the specific cell types being used (343). These AI-driven tools are anticipated to revolutionize device design, guiding the creation of an ideal technology tailored to individual patient needs. This integration of advanced computational techniques and artificial intelligence heralds a new era in tissue engineering, promising more effective and personalized solutions in the realm of beta cell replacement therapies.

## 5 Conclusion

In closing, Allo Beta Cell Transplantation represents a beacon of hope in the quest to transform the lives of individuals living with T1D. As we navigate the immunological intricacies that come with this therapeutic approach, innovation, collaboration, and a deep understanding of the interplay between the immune system and beta cells are the keys to success. With each unanswered question, we inch closer to effective solutions, and with each emerging strategy, we gain ground in the battle against T1D. As we move forward, we do so with a shared commitment to improving the lives of those who face the daily challenges of T1D, fuelled by the promise of Allo Beta Cell Transplantation and the resolve to conquer its immunological hurdles.

## Author contributions

RoC: Conceptualization, Writing – original draft. VT: Conceptualization, Writing – original draft. PMo: Conceptualization, Writing – original draft. VS: Conceptualization, Writing – original draft. AC: Conceptualization, Data curation, Writing – original draft. RaC: Writing – review & editing. CG: Writing – review & editing. DC: Writing – review & editing. ST: Writing – review & editing. VP: Writing – review & editing. RM: Writing – review & editing. AM: Writing – review & editing. RN: Writing – review & editing. PMa: Writing – review & editing. SP: Conceptualization, Writing – review & editing. LP: Conceptualization, Data curation, Writing – original draft, Writing – review & editing.
